# Temporal context modulates the recovery of the attentional blink

**DOI:** 10.1186/s41235-025-00625-6

**Published:** 2025-03-28

**Authors:** Fangshu Yao, Bin Zhou

**Affiliations:** 1 School of Psychology, Key Laboratory of Motor Cognitive Assessment and Regulation, Shanghai University of Sport, Shanghai, 200438 China; 2 State Key Laboratory of Cognitive Science and Mental Health, Institute of Psychology, Chinese Academy of Sciences, Beijing, 100101 China; 3 Department of Psychology, University of Chinese Academy of Sciences, Beijing, 101408 China

**Keywords:** Temporal context, Attentional blink, Random-walk, Interval timing, Attentional control

## Abstract

Humans usually adjust their attentional mode to tackle the challenges posed by environmental inputs. Depending on the uncertainty level, different attentional strategies may be adopted. As people face increasingly complicated daily situations—e.g., driving a car or chatting online—where intervals between significant events do not necessarily follow certain rules but are likely random, it appears important to understand how temporal contexts with different uncertainty levels affect temporal attention allocation when processing rapid serial inputs. We pursued this issue by employing a task examining the temporal limit of attention—the attentional blink (AB). The manipulation of temporal context was achieved by presenting trials with different inter-target intervals following either a “random-walk” or a “random” sequence. The results suggest a facilitated recovery from the AB deficit in the “random” compared to “random-walk” context, without a corresponding change in AB magnitude. Such effect is likely attributed to the higher perceived uncertainty in the former, and could be attenuated by a decrease in the temporal uncertainty level. These observations suggest that observers likely adopted a more flexible temporal attention allocation in the more unpredictable “random” context; they also support non-overlapping mechanisms responsible for AB width/duration and amplitude or lag-1 sparing. The flexibility of temporal attentional control may provide an evolutionary advantage for organisms to deal with unpredictable changes and is likely to be exploited for reference in the design of human–machine interacting platforms.

## Introduction

Temporal attention plays an important role in our daily life. For instance, the embedded temporal structure could be exploited to direct our attention to the most likely target moments to facilitate subsequent cognitive processing (Nobre & van Ede, 2018). In that way, drivers can avoid a potential collision and can orient their attention to relevant information. However, when two targets (T1 and T2) are embedded in a rapidly presented sequence of distractors, observers often miss T2 after correctly identifying T1 if T2 follows T1 within about half a second (Broadbent & Broadbent, 1987; Shapiro et al., 1997). This T2 deficit—known as the attentional blink (AB)—reflects the temporal limit of attending to two successive targets in close temporal vicinity (Raymond et al., 1992; Shapiro et al., 1997; Yao & Zhou, 2023), and thus provides an ideal platform to investigate the dynamics of temporal attention.

The AB is generally considered as a very robust phenomenon and is assumed by some researchers to arise because of a central bottleneck due to limited attentional resources (Chun & Potter, 1995; Duncan et al., 1994). However, pronounced individual differences and training effects (Nakatani et al., 2009; Willems & Martens, 2016) indicate that the AB is not so rigid as originally thought but sensitive to various trait and contextual factors. Moreover, the strict view of central bottleneck has been challenged by a growing number of studies (Di Lollo et al., 2005; Kawahara et al., 2006; Nieuwenstein, 2006; Nieuwenstein et al., 2005; Olivers & Nieuwenhuis, 2005, 2006; Olivers et al., 2007), which suggest that the dynamics of attentional allocation and its temporal constraint also play an important role in the generation of AB. Consistent with this, the AB effect has been posited as a multifaceted phenomenon with more than one locus of processing deficits (Willems & Martens, 2016; Zivony & Lamy, 2022) and being modulated by attentional control along the time dimension (Olivers, 2007; Wierda et al., 2012; Yao & Zhou, 2023). Oftentimes, the AB effect is mitigated by cognitive processes such as accurate temporal anticipation and proactive preparation for T2. For instance, knowing approximately at which lag (i.e., the time interval between targets) T2 will occur, either through statistical learning of lag probabilities, symbolic cueing of T2 lags, or even instantaneously processed lag information in a preceding trial, can alleviate the AB deficit (Choi et al., 2012; Hilkenmeier & Scharlau, 2010; Martens & Johnson, 2005; Visser et al., 2014; Yao et al., 2022). However, both the learning and exploitation of temporal regularities are likely to depend on the configuration of temporal context and the associated uncertainty level (Grabenhorst et al., 2021; Nobre & van Ede, 2018; Jazayeri & Shadlen, 2010; Shdeour et al., 2024). A similar dependence on the temporal configuration and its corresponding level of uncertainty can also be inferred in the AB task (Lasaponara et al., 2015). Somewhat counterintuitive, more distributed and flexible attentional control induced by task-irrelevant contexts could reduce the AB effect (Olivers & Nieuwenhuis, 2005, 2006). It thus appears that various temporal contexts may lead observers to employ distinct processing modes, such as a more focused attentional state in a predictable condition and a more flexible state in a highly uncertain condition, which ultimately shapes their performances in the AB task in a complementary manner. Though appealing in elucidating the mechanisms underlying AB, the role of the temporal context and related uncertainty level in the AB paradigm has been rarely explored and the existing evidence generally remains inconclusive (Dellert et al., 2022; Lasaponara et al., 2015). We therefore examined in the current study the role of temporal context, especially concerning the associated level of uncertainty/predictability, in the behavioral performance of the AB task.

Under natural situations, events usually develop in accord with a random-walk (Brownian) process, which is characterized by changes in an undetermined direction but with small steps, giving us a sense of continuity and stability. This is distinct from most laboratory settings (Glasauer & Shi, 2021; Narain et al., 2013), where stimuli are presented in a more unpredictable manner, with both changing size and changing direction randomly selected, leading to higher levels of perceived uncertainty. The differences between these two contexts could encourage individuals to differently exploit the available information and even adopt discrepant processing strategies, as would be implicated by views emphasizing the adaptive nature and performance-optimization properties of the neural system in coping with trial-to-trial variability (Jazayeri & Shadlen, 2010; Mamassian & Landy, 2010). We thus compared the AB effect between two temporal conditions where trials with different inter-target intervals (i.e., lags) unfolded following a random-walk and a random sequence.

Often demonstrated as a laboratory phenomenon, the AB has nevertheless a close link with some real-life scenarios, such as driving a car. Whereas our attention is continuously monitoring the road situation, a significant event (e.g., the changing behavior of the front car) may attract our attentional resources thus leading to temporary impairment of our ability to identify other potentially important events (e.g., a pedestrian rushes into the road). Critically, intervals between these events do not necessarily follow certain rules but are likely random, creating a context of high temporal uncertainty for us to cope with. Also relevant, in real-life face-to-face interactions, individuals may learn that their partner’s facial expression changes follow certain rules, both in terms of content and temporal intervals. However, with the development of online social media, the asynchrony of information transmission has been exaggerated (Ferguson et al., 2024), resulting in much higher temporal uncertainty (vs. live face-to-face communication), even when the interaction is aided by audiovisual technology. In this sense, a comparison of the AB effect under different temporal contexts could help to clarify how individuals regulate their attentional control along the time dimension when challenged by temporally uncertain inputs, thus providing potential insights into the design of platforms for human–machine interaction. In addition, considering that T2 performance gradually recovers toward long lags and is relatively spared at lag1 (i.e., the lag-1 sparing effect) (MacLean & Arnell, 2012) and that non-overlapping mechanisms are assumed to underlie these AB-related parameters (Cousineau et al., 2006; Livesey & Harris, 2011), we also examined whether the changing temporal context differently modulates AB amplitude, width/duration, minimum performance, and lag-1 sparing. Focusing on the factors modulating these effects can also help shed light on the underlying unresolved AB cognitive mechanisms.

## Experiment 1

The aim of Experiment 1 was to investigate the role of temporal context with different uncertainty levels in the behavioral performance of the AB task, focusing on AB amplitude, width/duration, minimum performance, and lag-1 sparing properties.

### Method

#### Participants

Thirty healthy young adults, naïve to the study, participated in Experiment 1 (18 females, *Mean age* ± *SD* = 20.4 ± 1.5 years). The sample size, based on an a priori power analysis using G*Power 3.1 software (Faul et al., 2007) with a medium-to-large effect size (*f* = 0.3) found in previous studies concerning the effect of task-irrelevant context on the AB effect (Arend et al., 2006; Olivers & Nieuwenhuis, 2005), was chosen to be larger than the recommended value (N = 24) that would result in sufficient power (≥ 0.8). All participants had normal or corrected-to-normal vision and were naïve to the purpose of the experiment. They gave informed consent before the experiment and were paid for their participation after the experiment. The procedure was approved by the Ethics Committee in Shanghai University of Sport (102772023RT041). Data were collected in 2023 and were statistically analyzed using JASP 0.17.1.0. The data that support the findings of this study are available in https://osf.io/ybrp5/. This study’s design and its analyses were not pre-registered.

#### Procedure

Visual stimuli were presented on a 24-inch LED monitor (60 Hz refresh rate, 1920 × 1080 pixel resolution) using the MATLAB R2016b software (®MathWorks) and the Psychophysics Toolbox extension (Brainard, 1997). Participants’ responses were collected by a standard keyboard. On each trial, a rapid serial visual presentation (RSVP) stream consisting of non-repeated letters was displayed in the center of the black screen. The letters were randomly selected from the English alphabet, except for E, O, I, and L, and were presented in Courier New Bold. Targets were presented in green while distractors were presented in white. Each item in the RSVP stream spanned a visual angle of ∼1.5° when viewed with a distance of 60 cm from the monitor.

**Fig. 1:**
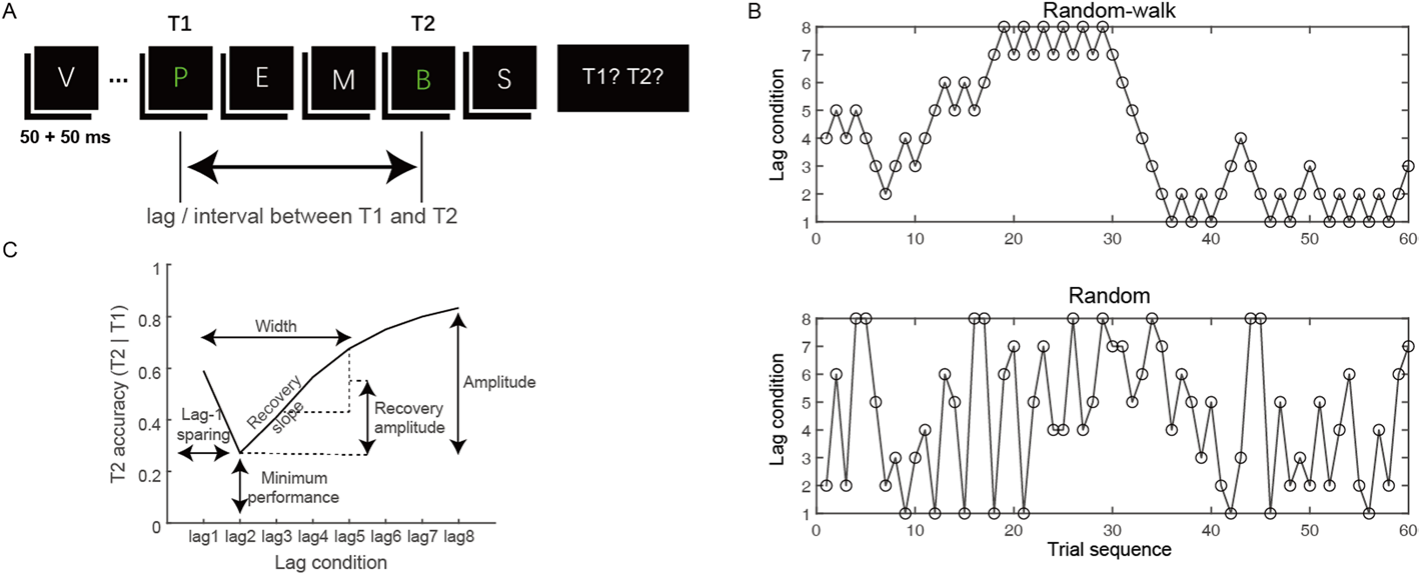
Experimental paradigm and stimuli. **A** Schematic representation of a single trial in the AB task. Participants were asked to report both targets which were separated by a given time interval in terms of lag. **B** An example of blocks of different temporal contexts (“random-walk” vs. “random”, 144 trials per block, only the first 60 trials are presented here), where the number of lag conditions was the same, but the order of trial sequences differed. **C** Schematic representation of the properties of the AB effect that were calculated in the current study

Each trial began with a white fixation cross in the center of the screen, and after a duration of 1000 ms, an RSVP stream of 18 letters was presented. Each item in the stream was presented for 50 ms, followed by a 50 ms blank interval, yielding an item presentation rate of 10 Hz (Fig. 1A). The first target (T1) was randomly presented at the fourth, fifth, or sixth position in the stream to avoid anticipation. The second target (T2) was presented at the first to eighth position after T1, referred to as lag1 to lag8, respectively. At the end of the RSVP stream, participants were required to report (or guess when not sure) the identity of both targets in the order of T1 then T2 by selecting from a 3 × 3 letter matrix presented in the center of the screen containing both targets and surrounding distractors which were most likely mis-selected as targets (Vul, Nieuwenstein, et al., ). Participants registered their responses with their right hand by pressing corresponding keys on the numeric keypad that spatially matched the selected letters in the letter matrix. Such a response mode simplified the procedure and made the responding process more straightforward. A new trial was initiated approximately 800–1000 ms after the registration of both responses. Participants practiced 24 trials in a random order before the experiment to familiarize themselves with the task. They then completed four randomly shuffled experimental blocks each containing 144 trials, with a break of at least three minutes long in between. Two blocks were “random-walk” blocks, in which trials with different lags, ranging from lag1 to lag8, were presented in a random-walk order. For example, the trial following lag3 was either lag4 (one step longer) or lag2 (one step shorter). The other two blocks were “random” blocks, where trials with different lags were randomly shuffled, but the trial number of each lag condition was kept identical to that in the corresponding “random-walk” block (Fig. 1B). Participants were not informed about the structure of trial sequences in different temporal contexts. Nevertheless, they could learn the structure (though likely implicitly) during the AB task. In each block, we ensured that the number of trials in each lag condition was at least 15.

#### Data analysis

Following the convention of AB studies, T1 and T2 accuracies were calculated as the percentage of correct T1 identifications and the percentage of correct T2 identifications given T1 being correctly reported, respectively. We first performed separate repeated-measures ANOVAs on T1 and T2 accuracies, with lag (lag1-8) and temporal context (“random-walk” vs. “random”) as within-subject factors. The Greenhouse–Geisser correction of the degrees of freedom was used whenever the sphericity assumption was violated. Because the AB effect is nonlinear and difficult to characterize (MacLean & Arnell, 2012), we also adopted the method of fitting our empirical data to the AB curve (mean *R*^2^ ≥ 0.88) to quantify its different aspects: lag-1 sparing,^1^ width, minimum performance, and amplitude (Cousineau et al., 2006). For the significant results observed in the AB recovery process, we additionally estimated the recovery amplitude by subtracting T2 accuracy at lag2 from the average at lag3-5, and the recovery slope by linearly fitting T2 accuracies across lag2-5 (Fig. 1C). We then compared these parameters across different temporal contexts in paired-sample t-tests.

### Results and discussion

**Fig. 2:**
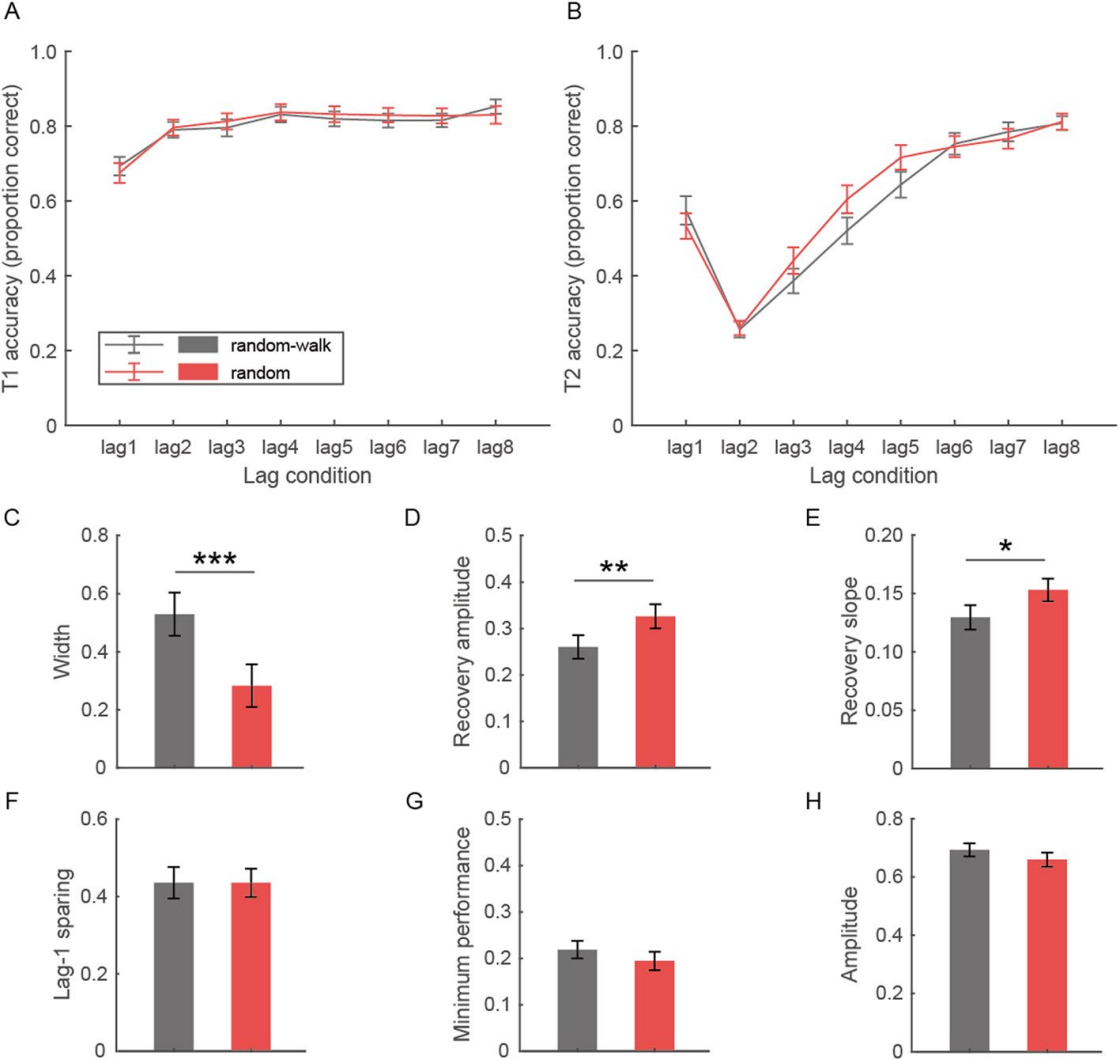
Behavioral results of the AB task in Experiment 1. **A**–**B** Accuracies in reporting T1 (**A**) and T2 (**B**) across lags in the “random-walk” and “random” conditions. **C**–**E** AB width and the recovery indices across temporal contexts. **F**–**H** The other AB properties across temporal contexts. Error bars represent standard errors (Barclay, 1979). * *p* < .05; ** *p* < .01; *** *p* < .001 in paired-sample t-tests

We first separately compared T1 and T2 accuracies between the “random-walk” and “random” conditions. As shown in Fig. 2A–B, the pattern of T1 and T2 accuracies changing with T1–T2 lags is in accord with observations of standard AB tasks. A repeated-measures ANOVA was performed on T1 accuracy, with lag and temporal context as within-subject factors. There was a main effect of lag (*F*(4.84, 140.40) = 34.36, *p* < 0.001, η_p_^2^ = 0.54), primarily driven by a significantly lower T1 accuracy at lag1 compared with other lags. For T2 performance, a similar 8 (lag) × 2 (temporal context) repeated-measures ANOVA was conducted. As expected, T2 accuracy varied significantly across lags (main effect of lag: *F*(3.30, 95.57) = 117.75, *p* < 0.001, η_p_^2^ = 0.80), exhibiting an AB effect together with lag-1 sparing. More interestingly, we found a significant main effect of temporal context (*F*(1, 29) = 4.59, *p* = 0.041, η_p_^2^ = 0.14), with a better performance in the “random” than “random-walk” condition. Such a contextual effect appeared restricted to several lags which lie within the AB recovery phase (lag × temporal context interaction: *F*(4.11, 119.05) = 3.51, *p* = 0.009, η_p_^2^ = 0.11; see lag3-5 from Fig. 2B).

To better quantify the properties of the AB effect, we estimated four parameters – the lag-1 sparing, width, minimum performance, and amplitude. Paired-sample t-tests were then conducted on these parameters to compare across temporal contexts. It turned out that only the AB width (*t*(29) = 4.32, *p* < 0.001, Cohen’s d = 0.79; Fig. 2C), but not the other characteristics (*p*s ≥ 0.26; Fig. 2F–H) of the AB curve, was modulated by temporal context. Since the AB width reflects the approximate duration of the AB window, this result suggests a significantly faster AB recovery in the “random” compared with “random-walk” temporal context. To confirm this effect, we calculated the recovery amplitude and recovery slope and then compared them in paired-sample t-tests. The results corroborated the observation that the “random” relative to “random-walk” temporal context facilitated the AB recovery (*t*(29) = 3.03, *p* = 0.005, Cohen’s d = 0.55 for the recovery amplitude; *t*(29) =  2.51, *p* = 0.018, Cohen’s d = 0.46 for the recovery slope; Fig. 2D–E).

In short, we observed a modulatory effect of temporal context on the AB effect at intermediate/long lags, although the most severe deficit lags remained unaffected. Such a facilitatory effect in the “random” relative to “random-walk” temporal context—appears to imply a faster AB recovery process—suggests that the sequential pattern of T1-T2 interval variation across trials, though largely task-irrelevant, alters the dynamics of resource allocation along the time dimension.

## Experiment 2

In Experiment 1, we observed a modulatory effect of temporal context (“random-walk” vs. “random”) on the AB curve, which could be attributed to differently perceived temporal uncertainties concerning lag intervals across trials and likely dissimilar ensuing processing strategies in these two temporal contexts. However, it remains to be determined whether participants were sensitive to the temporal information in these contexts, given that the identification of T2 was significantly affected when it appeared within the AB window. To address this issue, we conducted Experiment 2 and asked participants to estimate the perceived interval between two targets, in addition to the AB task, in each trial under both “random-walk” and “random” contexts. A secondary aim of Experiment 2 was to examine whether we could replicate the result pattern of Experiment 1 in a further group of participants with additional task demands.

### Method

#### Participants

We recruited another 30 participants (20 females, 20.6 ± 2.4 years) in Experiment 2. They were naïve to the purpose of the experiment and none of them participated in Experiment 1. All participants had normal or corrected-to-normal vision. All participants gave informed consent before the experiment and were paid for their participation after the experiment.

#### Procedure

The procedure of Experiment 2 was identical to that of Experiment 1, except that after reporting T1 and T2 in each trial, participants were additionally asked the question “How long do you think the interval between the two green targets was in the current trial”. They could choose from the following alternatives displayed below the question by pressing corresponding keys on the numeric keypad: “see only one target”, “relatively short”, “medium”, and “relatively long”, with their values ranging from 1 to 4.

#### Data analysis

We estimated a temporal sensitivity index (i.e., the slope) by linearly fitting the averaged ratings of the lag intervals across the lag1-8 conditions per participant. This index was then analyzed in one-sample t-tests (vs. 0) and paired-sample t-tests (“random-walk” vs. “random”). In addition, we also analyzed target accuracies (T1 and T2), model-estimated AB properties (lag-1 sparing, width, minimum performance, and amplitude), and another two AB recovery indices (recovery amplitude and recovery slope), in the same manner as in Experiment 1. To examine the difference between Experiments 1 and 2, we further performed separate mixed ANOVAs for T1 and T2 accuracies, with lag and temporal context as within-subject factors and experiment as the between-subject factor. We also analyzed all the AB-related properties with temporal context as the within-subject factor and experiment as the between-subject factor. Significant interactions between temporal context and experiment were further analyzed with simple main effects tests. The Greenhouse–Geisser correction of the degree of freedom was used whenever the sphericity assumption was violated.

### Results and discussion

**Fig. 3:**
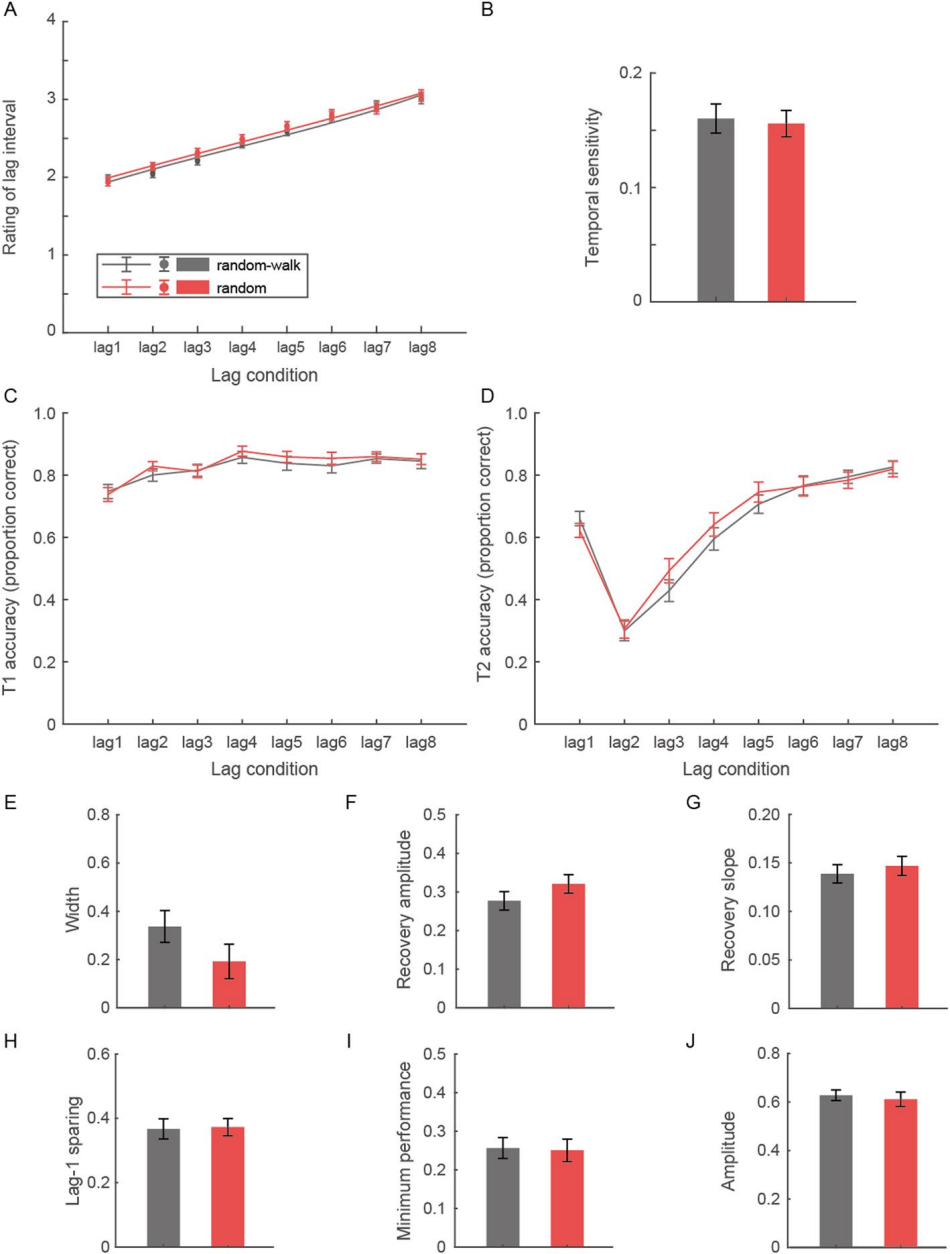
Subjective ratings and behavioral results of the AB task in Experiment 2. **A** Subjective ratings of the lag intervals across different temporal contexts. **B** Linear fit results of the rating scores across temporal contexts. **C**–**D** Accuracies in reporting T1 (**C**) and T2 (**D**) across lags in the “random-walk” and “random” conditions. **E**–**G** AB width and the recovery indices across temporal contexts. **H**–**J** The other AB properties across temporal contexts. Error bars represent standard errors

Following our primary purpose of the experiment, we examined the ratings of the lag interval between T1 and T2 in conditions of “random-walk” and “random” temporal contexts. As shown in Fig. 3A, the rating score increased with the increase of the lag interval, indicating that participants could generally discriminate the lag conditions in terms of T1–T2 interval duration. In support of this, the temporal sensitivity index (i.e., the slope) was significantly greater than 0 in all conditions (one-sample t-tests, *p*s < 0.001, Cohen’s ds ≥ 2.29, Fig. 3B). However, the temporal sensitivity index did not differ across temporal contexts (*t*(29) = 0.54, *p* = 0.59), suggesting that participants could perceive T1–T2 intervals comparably well in these two contextual conditions. These results indicate that participants were able to form the temporal gradient of different lag conditions in both temporal contexts despite their significantly impaired identification of T2 during AB, consistent with previous observations (Yao et al., 2022).

We then examined the pattern of T1 and T2 performances (Fig. 3C–D) and conducted repeated-measures ANOVAs with lag and temporal context as within-subject factors. For both T1 and T2 accuracies, the main effect of lag was significant (T1: *F*(3.99, 115.60) = 21.68, *p* < 0.001, η_p_^2^ = 0.43; T2: *F*(4.10, 118.86) = 124.87, *p* < 0.001, η_p_^2^ = 0.81), again indicating a typical observation of AB tasks. The result pattern of Experiment 1 was almost replicated, as there was also a significant interaction between lag and temporal context for T2 accuracy (*F*(5.14, 149.04) = 2.38, *p* = 0.039, η_p_^2^ = 0.08; also see lag3-5 from Fig. 3D). We then examined the lag-1 sparing, width, minimum performance, and amplitude of the AB effect, as well as the other two AB recovery indices (i.e., recovery amplitude and recovery slope) in paired-sample t-tests. Although the differences did not reach significance, both AB width (*t*(29) = − 1.87, *p* = 0.071, Cohen’s d = − 0.34; Fig. 3E) and recovery amplitude (*t*(29) = 1.75, *p* = 0.091, Cohen’s d = 0.32; Fig. 3F) showed a tendency of better recovery of performance in the “random” condition, similar to the result pattern in Experiment 1.

To confirm that the temporal contextual effects between Experiments 1 and 2 were comparable, we additionally performed separate mixed ANOVAs on T1 and T2 accuracies, with lag and temporal context as within-subject factors and experiment as the between-subject factor. No significant effects involving temporal context were ever found for T1 accuracy (*p*s ≥ 0.17) but there was a significant main effect of temporal context for T2 accuracy (*F*(1, 58) = 4.83, *p* = 0.032, η_p_^2^ = 0.08). There was also a significant interaction between lag and temporal context (*F*(5.29, 307.00) = 5.67, *p* < 0.001, η_p_^2^ = 0.09) for T2 accuracy, suggesting a better T2 performance in the "random" condition in both experiments, especially at those lags of the AB recovery period. Crucially, no significant interactions between temporal context and experiment were observed for both T1 and T2 accuracies (*p*s ≥ 0.45), indicating that the temporal contextual effects did not differ in the two experiments. Similar mixed ANOVA analyses with temporal context as the within-subject factor and experiment as the between-subject factor were also conducted on lag-1 sparing, width, minimum performance, amplitude, recovery amplitude, and recovery slope. Again, there were only significant main effects of temporal context for AB width (*F*(1, 58) = 16.63, *p* < 0.001, η_p_^2^ = 0.22), recovery amplitude (*F*(1, 58) = 10.91, *p* = 0.002, η_p_^2^ = 0.16), and recovery slope (*F*(1, 58) = 5.04, *p* = 0.029, η_p_^2^ = 0.08), but no significant interactions between temporal context and experiment for any of the parameters (*p*s ≥ 0.28), corroborating the evidence of comparable contextual effects between experiments. We have found in a previous study that explicitly estimating the T1–T2 interval might facilitate observers to exploit the local temporal information (e.g., the interval of an immediately preceding trial), which would benefit more for the “random-walk” compared to “random” condition (Yao et al., 2022). However, the results here suggested that the global temporal context with its associated uncertainty level likely played a more dominant role and regulated the dynamics of overall resource allocation along the time dimension, resulting in a likely facilitated recovery process in the “random” relative to “random-walk” temporal context.

## Experiment 3

The temporal uncertainty in the current study primarily arises from the changing direction and changing size of T1–T2 lags between successive trials. While the changing direction was uncertain in both “random-walk” and “random” conditions, the changing size was constant (one lag) in the former but varied substantially (from 0 to 7 lags) in the latter. Given that the perceptual uncertainty is highly related to the stimulus variation range and the probability of event occurrence (Spence et al., 2018; Trillenberg et al., 2000), it is conceivable that restricting the lag variation in the “random” condition would reduce the temporal uncertainty associated with the changing size and thus decrease the difference in temporal uncertainty between the above two conditions. Moreover, the timing uncertainty of T2 targets outside the AB window could profoundly affect the T2 performance within the AB window (Lasaponara et al., 2015). Given that Experiment 2 essentially replicated the result pattern of Experiment 1, we reduced the range of lags to 5 in Experiment 3 to examine whether such a manipulation, with the other aspects of the experimental design identical to Experiment 1, would result in a reduced temporal contextual effect supposedly associated with the level of temporal uncertainty.

### Method

#### Participants

In Experiment 3, we recruited 30 more participants (15 females, 20.5 ± 2.1 years, five of them also participated in Experiment 1). All participants had normal or corrected-to-normal vision. They were naïve to the purpose of the experiment. All participants gave informed consent before the experiment and were paid for their participation after the experiment.

#### Procedure

In Experiment 3, the range of inter-target lags was reduced to 5 (i.e., lag1-5), while the rest of the procedure was the same as in Experiment 1.

#### Data analysis

Similar to Experiments 1 and 2, we performed separate repeated-measures ANOVAs on T1 and T2 accuracies, with lag and temporal context as within-subject factors, and paired-sample t-tests on all AB-related properties (lag-1 sparing, width, minimum performance, amplitude, recovery amplitude, and recovery slope). To compare the difference between Experiments 1 and 3 and between Experiments 2 and 3, mixed ANOVAs were conducted for T1 and T2 accuracies with lag (only using data from lag1 to lag5) and temporal context as within-subject factors and experiment as the between-subject factor. We also analyzed the aforementioned AB parameters in mixed ANOVAs with temporal context as the within-subject factor and experiment as the between-subject factor. Significant interactions between temporal context and experiment were further analyzed with simple main effects tests. The Greenhouse–Geisser correction of the degrees of freedom was used whenever the sphericity assumption was violated.

### Results and discussion

**Fig. 4:**
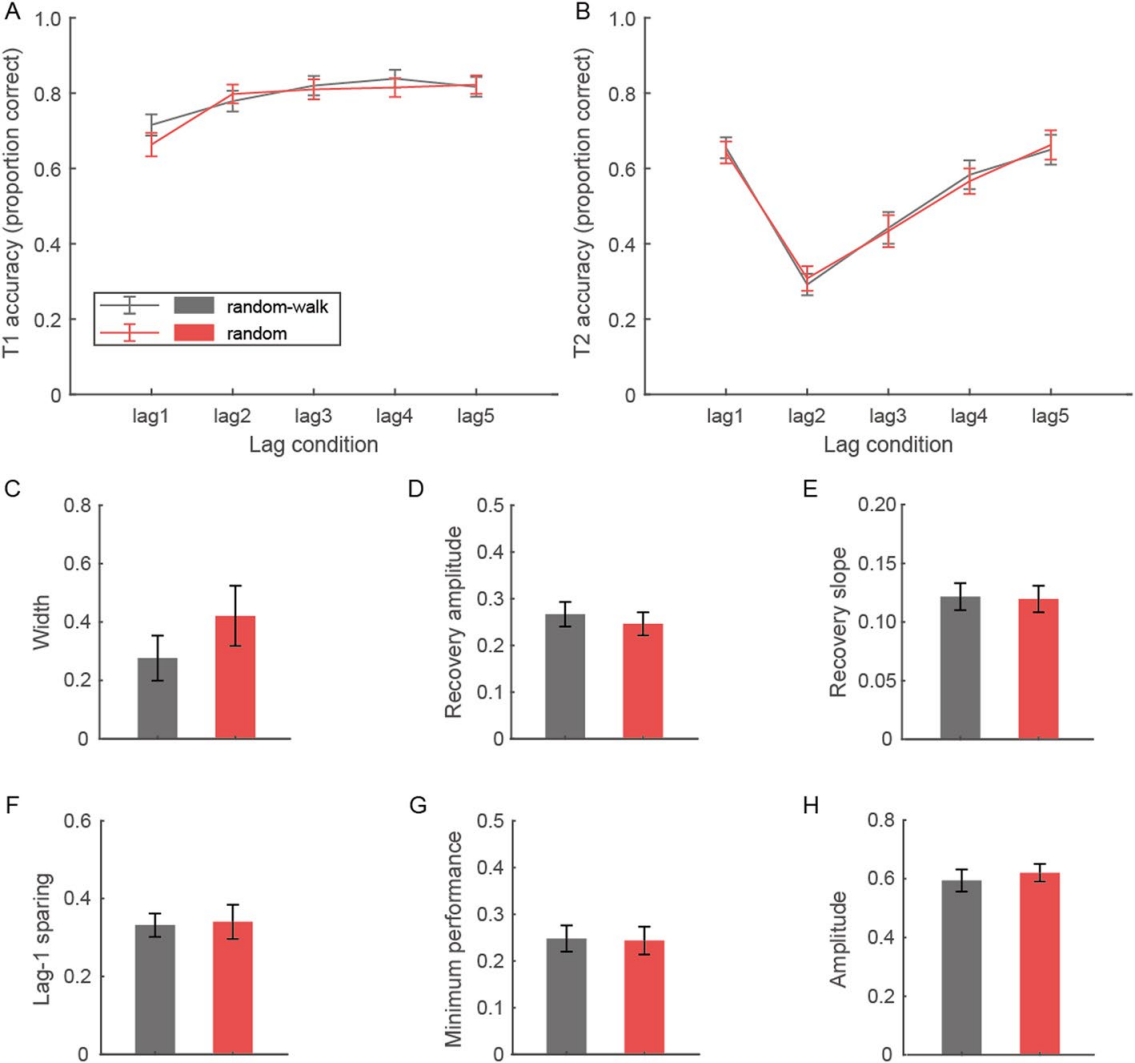
Behavioral results of the AB task in Experiment 3. **A**–**B** Accuracies in reporting T1 and T2 across lags in the “random-walk” and “random” conditions. **C**–**E** AB width and the recovery indices across temporal contexts. **F**–**H** The other AB properties across temporal contexts. Error bars represent standard errors

We first analyzed T1 and T2 accuracies in a repeated-measures ANOVA, respectively, with lag and temporal context as within-subject factors (Fig. 4A–B). For both targets, the main effects of lag (T1: *F*(3.12, 90.37) = 40.67, *p* < 0.001, η_p_^2^ = 0.58; T2: *F*(2.63, 76.20) = 50.22, *p* < 0.001, η_p_^2^ = 0.63) were significant. Importantly, we found no significant interaction between lag and temporal context for T2 performance (*F*(3.46, 110.36) = 0.55, *p* = 0.67). Paired-sample t-tests were conducted on AB-related parameters and no significant difference across temporal context was ever found (*p*s ≥ 0.19), suggesting that the pattern of the AB effect was largely identical between the two temporal contexts, either in terms of AB recovery or the other indices (Fig. 4C–H).

We then conducted mixed ANOVAs for T1 and T2 accuracies (lag × temporal context × experiment) and AB-related parameters (temporal context × experiment) to compare the temporal contextual effects between Experiments 1 and 3.^2^ The analysis on T1 did not show significant interaction between temporal context and experiment (*F*(1, 58) = 1.31, *p* = 0.26). In contrast, there was a significant temporal context × experiment interaction (*F*(1, 58) = 4.14, *p* = 0.047, η_p_^2^ = 0.07) and a significant three-way interaction (*F*(3.07, 178.26) = 2.65, *p* = 0.049, η_p_^2^ = 0.04) for T2 accuracy, although the main effect of experiment was not significant (*F*(1, 58) = 0.66, *p* = 0.42). The follow-up simple main effects analysis indicates a significant main effect of temporal context (i.e., higher T2 accuracy in the “random” relative to “random-walk” condition) in Experiment 1 (*F*(1, 29) = 11.45, *p* = 0.002, η_p_^2^ = 0.28) but not in Experiment 3 (*F*(1, 29) = 0.01, *p* = 0.91). We also observed a significant temporal context × experiment interaction for AB width (*F*(1, 58) = 10.39, *p* = 0.002, η_p_^2^ = 0.15), recovery amplitude (*F*(1, 58) = 9.23, *p* = 0.004, η_p_^2^ = 0.14), and a similar trend for recovery slope (*F*(1, 58) = 3.97, *p* = 0.051, η_p_^2^ = 0.06) but not for the other parameters (*p*s ≥ 0.24), indicating a significant temporal contextual effect in Experiment 1 but not in Experiment 3. In addition, this result pattern was not altered even when the five participants who participated in both experiments were excluded from the analysis. Together, the results of Experiment 3 suggest that the temporal contextual effect of AB is lag-range dependent and the associated temporal uncertainty is likely a substantial factor, if not the only one, modulating the AB recovery course observed in Experiments 1 and 2.

## General discussion

In this study, we manipulated the relationship of T1-T2 target intervals (i.e., lags) across successive trials according to either a random-walk (Brownian) or a random sequence and examined the consequent AB effect. The results primarily showed higher T2 performances at intermediate/long lags in the “random” compared with “random-walk” temporal context (Experiments 1 and 2), although the AB magnitude remained unaffected. Such results suggest a likely faster recovery of the AB deficit—as could be inferred from the smaller AB width, larger recovery amplitude, and steeper recovery slope—in the “random” temporal context. However, other processes leading to higher T2 performances at intermediate/long lags in the “random” condition are also possible (e.g., the dynamic balance between neural suppression and facilitation which may be not associated with attentional resource allocation) and cannot be definitely ruled out, which needs further clarification in future research. When the difference in the temporal uncertainty level was reduced, the contextual effect was abolished (Experiment 3). Given that the AB width (or duration, mainly reflecting the recovery process) is closely associated with the temporal dynamics of attentional control along the time dimension (MacLean & Arnell, 2012; Yao & Zhou, 2023), these results suggest that the overall temporal uncertainty context likely alters the cognitive strategy governing the temporal flexibility of allocating attentional resources.

As is common in the fields of physics, economics, and biology, the change—e.g., in particle movements, asset prices, and animal foraging patterns—is usually gradual and follows the random-walk procedure (Shi, 2024). As each movement or state transition depends on the previous one, this logical relationship of changes provides an ostensibly continuous sequential context and stability, seldom with abrupt changes of large steps or rules. Our neural system may exploit such a sequential property to simply follow its operation used in the preceding trial (i.e., local priors in terms of Bayesian inference) to optimize subsequent attentional deployment, as suggested by the probabilistic and computational view of neural functions (Ma & Jazayeri, 2014; Pouget et al., 2013). However, we also frequently encounter situations where the temporal change differences are large (e.g., following random sequences); this is particularly so in dynamic and complex situations. For instance, in driving scenarios, the intervals between successive critical events—e.g., a car in front brakes and a pedestrian rushes into the lane—are often unpredictable and vary largely from time to time. Similarly, in digital communication, messages from different partners are usually asynchronous with delays changing substantially from moment to moment. This can lead to significant temporal uncertainty. A strategy with flexible cognitive processes (e.g., flexible attentional allocation over time) appears more efficient to cope with such a stochastic situation. Indeed, flexibly changing the cognitive strategy according to the environmental state is common in the animal kingdom (Billard et al., 2020; Piet et al., 2018). Humans also adopt strategies with a similar, high degree of flexibility, whether it involves learning sensorimotor relationships (Narain et al., 2013), anticipatory hand movements (Bruhn et al., 2014), or social interaction (van den Berg & Wenseleers, 2018). The benefits of temporal uncertainty for error monitoring and adaptation to temporal structures have been demonstrated in previous studies (Korolczuk et al., 2024; Shdeour et al., 2024). Specifically, temporal unpredictability has been shown to slow impulsive go actions in a “go/no-go” task and foster the development of new temporal expectations during perceptual learning of temporal information.

In our study, the experienced higher temporal uncertainty of across-trial lag intervals in the “random” (vs. “random-walk”) context is more analogous to perceived volatility (vs. expected uncertainty) which may lead to enhanced change detection, frequent rule updating, and flexible decision-making (Bland & Schaefer, 2012; Monosov, 2020; Soltani & Izquierdo, 2019). It could signal participants to adopt a strategy to allocate attention along the time dimension more readily or flexibly. According to the two-stage model of the AB (Chun & Potter, 1995), the blinked T2 receives normal perceptual processing but is prevented from accessing a second capacity-limited stage that is already occupied by T1 processing. As T1 processing requires time, the flexible attentional allocation will benefit T2 performance most at intermediate/long lags residing on the AB recovery phase when T1 is finished or nearly finished. In addition, the more flexible temporal attention allocation in the “random” condition appears more globally structured. That is, the attentional resources in this condition may be distributed along a broader time range in a given trial, considering that T2 will occur at any possible lag. By comparison, the attentional resources in the “random-walk” condition can be allocated on the lag(s) similar to that of the preceding trial, thus more locally distributed. The globally structured attentional strategy (along the time dimension) may also contribute to the facilitated recovery of the AB deficit (or equally speaking, enhanced T2 performances at intermediate/long lags) in the “random” relative to “random-walk” condition.

It is worthy of noting that previous research has also indicated a facilitatory effect of temporal expectation on the performance of T2 in the AB task (Shen & Alain, 2012; Tang et al., 2014; Visser et al., 2015). More specifically, a delayed attentional engagement for T2 is assumed to be associated with the generation of the AB deficit (Nieuwenstein, 2006; Nieuwenstein et al., 2005), and pre-cueing the occurrence of T2 could improve the performance on T2 and thus ameliorate the AB effect (Martens & Johnson, 2005; Visser et al., 2014). Even unrelated immediately preceding temporal information (e.g., T1–T2 interval in the preceding trial) can be exploited to aid the report of T2 (Yao et al., 2022). As a result, one could expect that the AB effect would be reduced in the “random-walk” relative to “random” condition, given that the inter-target lag was more predictable under the “random-walk” context. However, we observed the opposite pattern of the AB effect, i.e., faster recovery of T2 performance in the “random” compared with “random-walk” condition. Such results might be accounted for by the different attentional strategies employed by participants in these two temporal contexts. Due to the lower level of temporal uncertainty in the “random-walk” condition, participants were more likely to adopt a focused attention strategy to use information of local preceding T1-T2 intervals to guide—to a certain extent—their temporal attention to a potential time point anticipating the occurrence of T2. This could facilitate the identification of T2 if it occurred at the expected time point, but make the control of attention less flexible to either disengage from the processing of T1 or reorient to T2 if it does not occur at the expected time point (Denison, 2024; Duyar et al., 2024; Yao & Zhou, 2023). The latter case would be frequent even in the “random-walk” context. In a sense, the disadvantage of focusing attention too much on a specific time point might override the benefit of temporal expectancy in certain temporal contexts. Consistent with this speculation, there was a trend suggesting that participants performed worse on trial N when the lag of trial N-2 was more deviant (lag deviant = 2 vs. lag deviant = 0; *t*(28) = -1.99, *p* = 0.056, Cohen’s d = 0.37) in the “random-walk” condition of Experiment 1. No such trend was observed in Experiments 2 and 3 (*p*s ≥ 0.55), nor in the “random” condition of all three experiments (*p*s ≥ 0.31). The lack of a trend in the “random-walk” condition in Experiment 2 was likely due to the additional timing demand for estimating the T1-T2 interval in each trial which attenuated the effect from trials beyond the immediately preceding one. Indeed, there appears a trade-off between focused and flexible attentional allocations in time, with the adoption of each strategy depending on the uncertainty/predictability of the temporal context. When the temporal uncertainty of the situation is high, as in the “random” condition of Experiment 1, individuals may tend to adopt a more flexible attentional control strategy over a broader time range. However, when the uncertainty in the “random” condition decreases or when the predictability of the “random-walk” condition increases—through a reduction in the variation range of the “random” condition or a slowing down of the changing pace in the “random-walk” condition—participants may shift to more focused attention on a certain lag to better perform the T2 report. As a result, the effect observed in Experiment 1 was either eliminated (in Experiment 3) or may even be reversed. It is currently unclear how precise the temporal predictability should be to outperform the facilitatory effect of flexible attentional allocation in AB performance or what is the optimal level of temporal uncertainty/predictability. This is an important factor and should be clarified in future research. Parametrically slowing down the random-walk pace (e.g., change once every 10 rather than 1 trials) appears a promising avenue.

Although no consensus has been reached concerning the exact mechanisms for the AB phenomenon, it is increasingly accepted that temporal attentional control plays an important role (Olivers, 2007; Yao & Zhou, 2023; Zivony & Lamy, 2022). A striking and counterintuitive observation reported in the literature indicates that the AB deficit is alleviated if less attention is focused on the processing of the target sequence (Olivers & Nieuwenhuis, 2005, 2006). It is argued that the AB deficit may result, at least partly, from the overinvestment of attentional resources in T1 and/or following distractors, which reduces the flexibility of attentional allocation on T2. The behaviors of temporal attention involved in the AB also contain suppressed selection and diffusion over time (Goodbourn et al., 2016; Vul, Hanus, et al., 2008a; Vul, Nieuwenstein, et al., 2008b), as well as a temporary loss then regaining control of an input filter configured by the endogenous attentional set (Di Lollo et al., 2005; Kawahara et al., 2006). Despite taking various forms, these empirical observations and theoretical accounts all suggest that it is the dynamics of temporal attentional control that form the core of mechanisms responsible for the AB deficit. Our observation is well in line with these hypothesized mechanisms.

Unlike most AB studies which observed modulations primarily on the minimum performance and amplitude of the AB effect (MacLean & Arnell, 2012), we found here that the duration/width of the AB effect can be independently modulated by the temporal uncertainty context, while the other characteristics of the AB effect remain unchanged. It is well in line with previous findings that the AB width/duration and the AB amplitude may reflect different components of the AB effect and can be modulated by different factors (Eich & Beck, 2023; Rizzo et al., 2001). Our findings further suggest that the temporal uncertainty may primarily affect the temporal dynamics of attentional allocation, rather than the amount of attentional resources which is likely associated with AB magnitude. These observations also add evidence to the view that the AB phenomenon is a multifaceted phenomenon that presumably arises from a combination of factors, including the attentional control along the temporal dimension, the details of which await further investigation.

Equally important yet unexplored are the distinct neural mechanisms underlying temporal contexts with varying levels of uncertainty. The perception of different types of uncertainty is hierarchically encoded by the human cortex (Diaconescu et al., 2017; Iglesias et al., 2013) and involves widespread brain areas, particularly the frontoparietal network. Research on primates has shown that higher levels of contextual uncertainty selectively enhance the information transmission from the parietal to the frontal lobe while suppressing the transmission in the opposite direction (Taghizadeh et al., 2020). This is in line with the Bayesian learning principle, which suggests that bottom-up sensory inputs (as opposed to top-down priors) are prioritized when the level of prior uncertainty is high (Mathys et al., 2011). Such cortical circuits oppositely regulating the bottom-up and top-down signals may provide a promising neural candidate underlying the temporal contextual effect observed in our study. We speculate that the “random-walk” condition set the immediate priors (i.e., the lag interval in the preceding trial; via frontal-to-parietal transmission) dominant in determining the temporal attention allocation in the current trial, whereas the “random” condition relied more on new sensory inputs to generate posteriors (via parietal-to-frontal transmission) and drove temporal attention allocation in a more bottom-up and flexible manner. Nevertheless, further research is needed to delineate the involved neural mechanisms underlying our observation here.

In sum, our result suggests a likely accelerated AB recovery process when participants were confronted with a more unpredictable environment. In such a situation, participants were unable to use local temporal information to anticipate the upcoming events and thus adopted a cognitive strategy with more flexible temporal attentional control, which led to a less pronounced AB deficit at intermediate/long lags. These results suggest that the temporal limitations in our attentional resource allocation can be modulated by the perceived level of temporal uncertainty/predictability. They may be also informative in developing better human–machine interfaces such as those utilized for driving and online communication. In these scenarios, information may be delivered in forms of continuous streams on the display platforms, with successive critical messages (e.g., social signals from different communicating partners) sometimes presented in the time range consistent with intermediate/long lags in the AB task. To reduce the impact of consciously detecting one target on the detection of subsequent targets, one may manipulate the temporal context of inter-target intervals to find the optimal level of cognitive strategy.

## Data Availability

The data that support the findings of this study are available in https://osf.io/ybrp5/.

## Footnotes

1 We also calculated the lag-1 sparing in a traditional way by the performance difference between lag1 and lag2 (Livesey & Harris, 2011). None of the lag-1 sparing effects were significantly different between “random-walk” and “random” conditions across three experiments (*p*s ≥ .13).

2 Similar mixed ANOVAs were performed to compare the temporal contextual effects on T1 and T2 accuracies as well as AB-related parameters across Experiment 2 and Experiment 3. Although the interaction between temporal context and experiment was not significant for T1 and T2 accuracies (*p*s ≥ 0.13), a significant interaction was observed for AB width (*F*(1, 58) = 4.80, *p* = 0.032, η_p_^2^ = 0.08) and recovery amplitude (*F*(1, 58) = 4.29, *p* = 0.043, η_p_^2^ = 0.07), suggesting a different pattern of AB recovery in Experiment 2 relative to Experiment 3.

## References

[CR1] Arend, I., et al. (2006). Task-irrelevant visual motion and flicker attenuate the attentional blink. *Psychonomic Bulletin & Review,**13*(4), 600–607. 10.3758/BF03193969 17201358 10.3758/bf03193969

[CR2] Barclay, W. R. (1979). Statistics for the biological sciences. *JAMA: the Journal of the American Medical Association,**241*(21), 2315. 10.1001/jama.1979.03290470063037

[CR3] Billard, P., et al. (2020). Cuttlefish show flexible and future-dependent foraging cognition. *Biology Letters,**16*(2), 20190743. 10.1098/rsbl.2019.0743 32019464 10.1098/rsbl.2019.0743 PMC7058941

[CR4] Bland, A. R., & Schaefer, A. (2012). Different varieties of uncertainty in human decision-making. *Frontiers in Neuroscience,**6*, 85. 10.3389/fnins.2012.00085 22701401 10.3389/fnins.2012.00085 PMC3370661

[CR5] Brainard, D. H. (1997). The psychophysics toolbox. *Spatial Vision,**10*(4), 433–436. 10.1163/156856897X00357 9176952

[CR6] Broadbent, D. E., & Broadbent, M. H. P. (1987). From detection to identification: Response to multiple targets in rapid serial visual presentation. *Perception & Psychophysics,**42*(2), 105–113. 10.3758/BF03210498 3627930 10.3758/bf03210498

[CR7] Bruhn, P., et al. (2014). Degree of certainty modulates anticipatory processes in real time. *Journal of Experimental Psychology: Human Perception and Performance,**40*(2), 525–538. 10.1037/a0034365 24041331 10.1037/a0034365

[CR8] Choi, H., et al. (2012). Resetting capacity limitations revealed by long-lasting elimination of attentional blink through training. *Proceedings of the National Academy of Sciences of the United States of America*, 109(30), 12242-12247. 10.1073/pnas.1203972109 22778408 10.1073/pnas.1203972109 PMC3409736

[CR9] Chun, M. M., & Potter, M. C. (1995). A two-stage model for multiple target detection in rapid serial visual presentation. *Journal of Experimental Psychology: Human Perception and Performance,**21*(1), 109. 10.1037/0096-1523.21.1.109 7707027 10.1037//0096-1523.21.1.109

[CR10] Cousineau, D., et al. (2006). Parameterizing the attentional blink effect. *Canadian Journal of Experimental Psychology,**60*(3), 175–189. 10.1037/cjep2006017 17076433 10.1037/cjep2006017

[CR11] Dellert, T., et al. (2022). Neural correlates of consciousness in an attentional blink paradigm with uncertain target relevance. *NeuroImage,**264*, 119679. 10.1016/j.neuroimage.2022.119679 36220535 10.1016/j.neuroimage.2022.119679

[CR12] Denison, R. N. (2024). Visual temporal attention from perception to computation. *Nature Reviews Psychology,**3*, 261–274. 10.1038/s44159-024-00294-0

[CR13] Di Lollo, V., et al. (2005). The attentional blink: Resource depletion or temporary loss of control? *Psychological Research Psychologische Forschung,**69*(3), 191–200. 10.1007/s00426-004-0173-x 15597184 10.1007/s00426-004-0173-x

[CR14] Diaconescu, A. O., et al. (2017). A computational hierarchy in human cortex. arXiv preprint arXiv:1709.02323. 10.48550/arXiv.1709.02323

[CR15] Duncan, J., et al. (1994). Direct measurement of attentional dwell time in human vision. *Nature,**369*(6478), 313–315. 10.1038/369313a0 8183369 10.1038/369313a0

[CR16] Duyar, A., et al. (2024). When temporal attention interacts with expectation. *Scientific Reports,**14*(1), 4624. 10.1038/s41598-024-55399-6 38409235 10.1038/s41598-024-55399-6 PMC10897459

[CR17] Eich, B., & Beck, M. R. (2023). Differences in the duration of the attentional blink when viewing nature vs. urban scenes. *Attention, Perception, & Psychophysics,**85*(6), 1846–1867. 10.3758/s13414-023-02749-7 10.3758/s13414-023-02749-7 37415062

[CR18] Faul, F., et al. (2007). G* Power 3: A flexible statistical power analysis program for the social, behavioral, and biomedical sciences. *Behavior Research Methods,**39*(2), 175–191. 10.3758/BF03193146 17695343 10.3758/bf03193146

[CR19] Ferguson, A. M., et al. (2024). Social uncertainty in the digital world. *Trends in Cognitive Sciences,**28*(4), 286–289. 10.1016/j.tics.2024.02.005 38448356 10.1016/j.tics.2024.02.005 PMC10993016

[CR20] Glasauer, S., & Shi, Z. (2021). The origin of Vierordt’s law: The experimental protocol matters. *PsyCh Journal,**10*(5), 732–741. 10.1002/pchj.464 34028202 10.1002/pchj.464

[CR21] Goodbourn, P. T., et al. (2016). Reconsidering temporal selection in the attentional blink. *Psychological Science,**27*(8), 1146–1156. 10.1177/0956797616654131 27407133 10.1177/0956797616654131

[CR22] Grabenhorst, M., et al. (2021). Two sources of uncertainty independently modulate temporal expectancy. *Proceedings of the National Academy of Sciences of the United States of America*, *118*(16), e2019342118. 10.1073/pnas.2019342118 33853943 10.1073/pnas.2019342118 PMC8072397

[CR23] Nobre, A. C., & van Ede, F. (2018). Anticipated moments: temporal structure in attention. *Nature Reviews Neuroscience, 19*(1), 34–48. 10.1038/nrn.2017.141 29213134 10.1038/nrn.2017.141

[CR24] Hilkenmeier, F., & Scharlau, I. (2010). Rapid allocation of temporal attention in the attentional blink paradigm. *European Journal of Cognitive Psychology,**22*(8), 1222–1234. 10.1080/09541440903418924

[CR25] Iglesias, S., et al. (2013). Hierarchical prediction errors in midbrain and basal forebrain during sensory learning. *Neuron,**80*(2), 519–530. 10.1016/j.neuron.2013.09.009 24139048 10.1016/j.neuron.2013.09.009

[CR26] Jazayeri, M., & Shadlen, M. N. (2010). Temporal context calibrates interval timing. *Nature Neuroscience,**13*(8), 1020–1026. 10.1038/nn.2590 20581842 10.1038/nn.2590 PMC2916084

[CR27] Kawahara, J., et al. (2006). The attentional blink is governed by a temporary loss of control. *Psychonomic Bulletin & Review,**13*(5), 886–890. 10.3758/bf03194014 17328390 10.3758/bf03194014

[CR28] Korolczuk, I., et al. (2024). Temporal unpredictability increases error monitoring as revealed by EEG-EMG investigation. *Psychophysiology,**61*(2), e14442. 10.1111/psyp.14442 37724801 10.1111/psyp.14442

[CR29] Lasaponara, S., et al. (2015). The “serendipitous brain”: Low expectancy and timing uncertainty of conscious events improve awareness of unconscious ones (evidence from the Attentional Blink). *Cortex,**71*(2015), 15–33. 10.1016/j.cortex.2015.05.029 26142182 10.1016/j.cortex.2015.05.029

[CR30] Livesey, E. J., & Harris, I. M. (2011). Target sparing effects in the attentional blink depend on type of stimulus. *Attention, Perception, & Psychophysics,**73*(7), 2104–2123. 10.3758/s13414-011-0177-8 10.3758/s13414-011-0177-8 21751050

[CR31] Ma, W. J., & Jazayeri, M. (2014). Neural coding of uncertainty and probability. *Annual Review of Neuroscience,**37*(1), 205–220. 10.1146/annurev-neuro-071013-014017 25032495 10.1146/annurev-neuro-071013-014017

[CR32] MacLean, M., & Arnell, K. (2012). A conceptual and methodological framework for measuring and modulating the attentional blink. *Attention, Perception, & Psychophysics,**74*(6), 1080–1097. 10.3758/s13414-012-0338-4 10.3758/s13414-012-0338-4 22821263

[CR33] Mamassian, P., & Landy, M. S. (2010). It’s that time again. *Nature Neuroscience,**13*(2010), 914–916. 10.1038/nn0810-914 20661267 10.1038/nn0810-914 PMC4170728

[CR34] Martens, S., & Johnson, A. (2005). Timing attention: Cuing target onset interval attenuates the attentional blink. *Memory & Cognition,**33*(2), 234–240. 10.3758/BF03195312 16028578 10.3758/bf03195312

[CR35] Mathys, C. D., et al. (2011). A bayesian foundation for individual learning under uncertainty. *Frontiers in Human Neuroscience,**5*, 39. 10.3389/fnhum.2011.00039 21629826 10.3389/fnhum.2011.00039 PMC3096853

[CR36] Monosov, I. E. (2020). How outcome uncertainty mediates attention, learning, and decision-making. *Trends in Neurosciences,**43*(10), 795–809. 10.1016/j.tins.2020.06.009 32736849 10.1016/j.tins.2020.06.009 PMC8153236

[CR37] Nakatani, C., et al. (2009). Practice begets the second target: Task repetition and the attentional blink effect. *Progress in Brain Research,**176*, 123–134. 10.1016/S0079-6123(09)17608-2 19733753 10.1016/S0079-6123(09)17608-2

[CR38] Narain, D., et al. (2013). How the statistics of sequential presentation influence the learning of structure. *PLoS ONE,**8*(4), e62276. 10.1371/journal.pone.0062276 23638022 10.1371/journal.pone.0062276 PMC3634735

[CR39] Nieuwenstein, M. R., et al. (2005). Delayed attentional engagement in the attentional blink. *Journal of Experimental Psychology: Human Perception and Performance,**31*(6), 1463–1475. 10.1037/0096-1523.31.6.1463 16366802 10.1037/0096-1523.31.6.1463

[CR40] Nieuwenstein, M. R. (2006). Top-down controlled, delayed selection in the attentional blink. *Journal of Experimental Psychology: Human Perception and Performance,**32*(4), 973–985. 10.1037/0096-1523.32.4.973 16846292 10.1037/0096-1523.32.4.973

[CR41] Olivers, C. N., et al. (2007). Spreading the sparing: Against a limited-capacity account of the attentional blink. *Psychological Research Psychologische Forschung,**71*(2), 126–139. 10.1007/s00426-005-0029-z 16341546 10.1007/s00426-005-0029-z

[CR42] Olivers, C. N. (2007). The time course of attention: It is better than we thought. *Current Directions in Psychological Science,**16*(1), 11–15. 10.1111/j.1467-8721.2007.00466.x

[CR43] Olivers, C. N., & Nieuwenhuis, S. (2005). The beneficial effect of concurrent task-irrelevant mental activity on temporal attention. *Psychological Science,**16*(4), 265–269. 10.1111/j.0956-7976.2005.01526.x 15828972 10.1111/j.0956-7976.2005.01526.x

[CR44] Olivers, C. N., & Nieuwenhuis, S. (2006). The beneficial effects of additional task load, positive affect, and instruction on the attentional blink. *Journal of Experimental Psychology: Human Perception and Performance,**32*(2), 364–379. 10.1037/0096-1523.32.2.364 16634676 10.1037/0096-1523.32.2.364

[CR45] Piet, A. T., et al. (2018). Rats adopt the optimal timescale for evidence integration in a dynamic environment. *Nature Communications,**9*(1), 4265. 10.1038/s41467-018-06561-y 30323280 10.1038/s41467-018-06561-y PMC6189050

[CR46] Pouget, A., et al. (2013). Probabilistic brains: Knowns and unknowns. *Nature Neuroscience,**16*(9), 1170–1178. 10.1038/nn.3495 23955561 10.1038/nn.3495 PMC4487650

[CR47] Raymond, J. E., et al. (1992). Temporary suppression of visual processing in an RSVP task: An attentional blink? *Journal of Experimental Psychology: Human Perception and Performance,**18*(3), 849–860. 10.1037/0096-1523.18.3.849 1500880 10.1037//0096-1523.18.3.849

[CR48] Rizzo, M., et al. (2001). Increased attentional blink after focal cerebral lesions. *Neurology,**57*(5), 795–800. 11552006 10.1212/wnl.57.5.795

[CR49] Shapiro, K. L., et al. (1997). The attentional blink. *Trends in Cognitive Sciences,**1*(8), 291–296. 10.1016/s1364-6613(97)01094-2 21223931 10.1016/S1364-6613(97)01094-2

[CR50] Shdeour, O., et al. (2024). Exposure to temporal variability promotes subsequent adaptation to new temporal regularities. *Cognition,**244*, 105695. 10.1016/j.cognition.2023.105695 38183867 10.1016/j.cognition.2023.105695

[CR51] Shen, D., & Alain, C. (2012). Implicit temporal expectation attenuates auditory attentional blink. *PLoS ONE,**7*(4), e36031. 10.1371/journal.pone.0036031 22558312 10.1371/journal.pone.0036031 PMC3338751

[CR52] Shi, J. (2024). Exploring the meandering pathways of random walk: From finance to physics, biology, and beyond. *Highlights in Science, Engineering and Technology,**88*, 162–168. 10.54097/48kqs989

[CR53] Soltani, A., & Izquierdo, A. (2019). Adaptive learning under expected and unexpected uncertainty. *Nature Reviews Neuroscience,**20*(10), 635–644. 10.1038/s41583-019-0180-y 31147631 10.1038/s41583-019-0180-y PMC6752962

[CR54] Spence, M., et al. (2018). Uncertainty information that is irrelevant for report impacts confidence judgments. *Journal of Experimental. Psychology Human Perception and Performance,**44*(12), 1981–1994. 10.1037/xhp0000584 30475052 10.1037/xhp0000584

[CR55] Taghizadeh, B., et al. (2020). Reward uncertainty asymmetrically affects information transmission within the monkey fronto-parietal network. *Communications Biology,**3*(1), 594. 10.1038/s42003-020-01320-6 33087809 10.1038/s42003-020-01320-6 PMC7578031

[CR56] Tang, M. F., et al. (2014). Training and the attentional blink: Limits overcome or expectations raised? *Psychonomic Bulletin & Review,**21*(2), 406–411. 10.3758/s13423-013-0491-3 23884691 10.3758/s13423-013-0491-3

[CR57] Trillenberg, P., et al. (2000). CNV and temporal uncertainty with ‘ageing’ and ‘non-ageing’ S1–S2 intervals. *Clinical Neurophysiology,**111*(7), 1216–1226. 10.1016/S1388-2457(00)00274-1 10880797 10.1016/s1388-2457(00)00274-1

[CR58] van den Berg, P., & Wenseleers, T. (2018). Uncertainty about social interactions leads to the evolution of social heuristics. *Nature Communications,**9*(1), 2151. 10.1038/s41467-018-04493-1 29855472 10.1038/s41467-018-04493-1 PMC5981325

[CR59] Visser, T. A., et al. (2014). Temporal cues and the attentional blink: A further examination of the role of expectancy in sequential object perception. *Attention, Perception, & Psychophysics,**76*(8), 2212–2220. 10.3758/s13414-014-0710-7 10.3758/s13414-014-0710-7 24935807

[CR60] Visser, T. A., et al. (2015). Temporal cues derived from statistical patterns can overcome resource limitations in the attentional blink. *Attention, Perception, & Psychophysics,**77*(5), 1585–1595. 10.3758/s13414-015-0880-y 10.3758/s13414-015-0880-y 25813742

[CR61] Vul, E., et al. (2008a). Delay of selective attention during the attentional blink. *Vision Research,**48*(18), 1902–1909. 10.1016/j.visres.2008.06.009 18611406 10.1016/j.visres.2008.06.009 PMC2610420

[CR62] Vul, E., et al. (2008b). Temporal selection is suppressed, delayed, and diffused during the attentional blink. *Psychological Science,**19*(1), 55–61. 10.1111/j.1467-9280.2008.02046.x 18181792 10.1111/j.1467-9280.2008.02046.x PMC2744451

[CR63] Wierda, S. M., et al. (2012). Pupil dilation deconvolution reveals the dynamics of attention at high temporal resolution. *Proceedings of the National Academy of Sciences of the United States of America*, *109*(22), 8456–8460. 10.1073/pnas.1201858109 22586101 10.1073/pnas.1201858109 PMC3365158

[CR64] Willems, C., & Martens, S. (2016). Time to see the bigger picture: Individual differences in the attentional blink. *Psychonomic Bulletin & Review,**23*(5), 1289–1299. 10.3758/s13423-015-0977-2 26576803 10.3758/s13423-015-0977-2 PMC5050248

[CR65] Yao, F., et al. (2022). Immediate temporal information modulates the target identification in the attentional blink. *Brain Sciences,**12*(2022), 278. 10.3390/brainsci12020278 35204041 10.3390/brainsci12020278 PMC8870607

[CR66] Yao, F., & Zhou, B. (2023). It’s time for attentional control: Temporal expectation in the attentional blink. *Consciousness and Cognition,**107*(2023), 103461. 10.1016/j.concog.2022.103461 36584439 10.1016/j.concog.2022.103461

[CR67] Zivony, A., & Lamy, D. (2022). What processes are disrupted during the attentional blink? An integrative review of event-related potential research. *Psychonomic Bulletin & Review,**29*(2), 394–414. 10.3758/s13423-021-01973-2 34291430 10.3758/s13423-021-01973-2

